# NetB, a New Toxin That Is Associated with Avian Necrotic Enteritis Caused by Clostridium perfringens

**DOI:** 10.1371/journal.ppat.0040026

**Published:** 2008-02-08

**Authors:** Anthony L Keyburn, John D Boyce, Paola Vaz, Trudi L Bannam, Mark E Ford, Dane Parker, Antonio Di Rubbo, Julian I Rood, Robert J Moore

**Affiliations:** 1 CSIRO Livestock Industries, Australian Animal Health Laboratory, Geelong, Victoria, Australia; 2 Department of Microbiology, ARC Centre of Excellence in Structural and Functional Microbial Genomics, Monash University, Clayton, Victoria, Australia; 3 Australian Poultry Cooperative Research Centre, Armidale, New South Wales, Australia; University of California Los Angeles, United States of America

## Abstract

For over 30 years a phospholipase C enzyme called alpha-toxin was thought to be the key virulence factor in necrotic enteritis caused by Clostridium perfringens. However, using a gene knockout mutant we have recently shown that alpha-toxin is not essential for pathogenesis. We have now discovered a key virulence determinant. A novel toxin (NetB) was identified in a C. perfringens strain isolated from a chicken suffering from necrotic enteritis (NE). The toxin displayed limited amino acid sequence similarity to several pore forming toxins including beta-toxin from C. perfringens (38% identity) and alpha-toxin from Staphylococcus aureus (31% identity). NetB was only identified in C. perfringens type A strains isolated from chickens suffering NE. Both purified native NetB and recombinant NetB displayed cytotoxic activity against the chicken leghorn male hepatoma cell line LMH; inducing cell rounding and lysis. To determine the role of NetB in NE a *netB* mutant of a virulent C. perfringens chicken isolate was constructed by homologous recombination, and its virulence assessed in a chicken disease model. The *netB* mutant was unable to cause disease whereas the wild-type parent strain and the *netB* mutant complemented with a wild-type *netB* gene caused significant levels of NE. These data show unequivocally that in this isolate a functional NetB toxin is critical for the ability of C. perfringens to cause NE in chickens. This novel toxin is the first definitive virulence factor to be identified in avian C. perfringens strains capable of causing NE. Furthermore, the *netB* mutant is the first rationally attenuated strain obtained in an NE-causing isolate of C. perfringens; as such it has considerable vaccine potential.

## Author Summary

Clostridium perfringens can cause gas gangrene and food poisoning in humans and causes several enterotoxemic diseases in animals including avian necrotic enteritis. This disease affects all chicken producing countries worldwide and is a considerable burden on the commercial chicken production industry. Until recently alpha-toxin was thought to be the major virulence factor involved in necrotic enteritis. However, by using an alpha-toxin null mutant it has been demonstrated that this toxin is not essential for disease. This paper details the identification and characterisation of a novel toxin, NetB, and provides evidence that the protein is an essential factor in causing necrotic enteritis in chickens. NetB has limited protein sequence identity to the beta-toxin of C. perfringens, which causes mucosal necrosis of the small intestine in humans and animals. We demonstrate that NetB null mutants can no longer cause disease in chickens, whereas both the wild-type and mutant complemented with a wild-type *netB* gene caused significant levels of necrotic enteritis. The identification of this important toxin advances our understanding of the pathogenesis of the disease and opens significant opportunities for the development of novel vaccines against necrotic enteritis in poultry.

## Introduction

Clostridium perfringens is the main causative agent of avian necrotic enteritis (NE), an enteric disease of chickens that was first described in 1961 [1] and has since been found in all poultry producing countries. NE in chickens manifests as an acute or chronic enterotoxemia [2]. The acute disease results in significant levels of mortality whereas the chronic disease leads to loss of productivity and welfare concerns. It has been estimated that the disease costs the international poultry industry in excess of $US2 billion per year [3–5]. NE is primarily caused by C. perfringens type A and to a lesser extent type C strains. Clinical NE is thought to occur when C. perfringens proliferates to high numbers in the small intestine and produces extracellular toxins that damage the gastrointestinal tract.

Early studies on NE suggested that the main virulence factor involved in the disease was secreted by the bacteria [6], which led to the proposal that alpha-toxin was the major toxin involved in pathogenesis. This hypothesis was based on the observations that alpha-toxin is a major secreted protein and that cell-containing and cell-free cultures were able to cause necrotic lesions typical of NE in the gastrointestinal tract of chickens [6–8]. We have constructed an alpha-toxin (*plc*) null mutant of a virulent necrotic enteritis isolate, EHE-NE18, and have shown that it can still cause disease in chickens, proving that alpha-toxin is not an essential virulence factor and questioning the role of alpha-toxin in the disease process [9].

C. perfringens is a Gram-positive anaerobe and is ubiquitous in the environment, being found in the soil, in decaying organic matter and as a member of the normal intestinal flora of many humans and animals [10]. It has been implicated in numerous diseases [11]. C. perfringens strains produce many different secreted toxins including beta-toxin, a pore-forming toxin that is related to alpha-toxin from Staphylococcus aureus. The S. aureus alpha-toxin, which is not related to the C. perfringens alpha-toxin, forms functional oligomers in membranes and beta-toxin forms a heptameric oligomer on the surface of HL60 cells [12]. Beta-toxin has been implicated in diseases of both man and animals; these diseases are generally characterized by an acute onset with lethal hemorrhagic mucosal ulceration or severe mucosal necrosis of the small intestine [13].

In this study we report the identification and purification of a novel toxin, NetB, from C. perfringens, and show that it has limited similarity to C. perfringens beta-toxin. A *C. perfringens netB* mutant was constructed and found to be avirulent in an NE induction animal model; a process that was reversed when the mutation was complemented by the wild-type *netB* gene. Therefore, the results show that NetB is a critical virulence factor in the pathogenesis of NE in chickens. The *netB* mutant represents the first example of a rationally attenuated strain obtained by direct targeting of a specific virulence factor of chicken-derived strains of C. perfringens.

## Results

### EHE-NE18 Culture Supernatant Is Cytotoxic to LMH Cells

**Figure 1:**
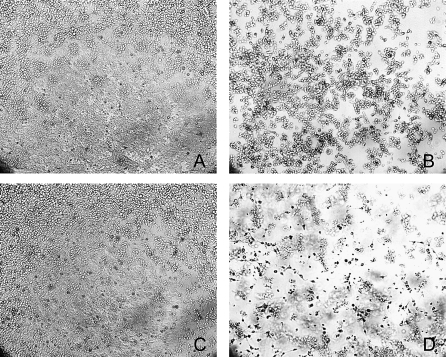
Cytotoxic Activity of EHE-NE18 Supernatant on LMH Cells The LMH cells were grown to 70% confluence and culture supernatant added to the medium as a 2-fold dilution series across the plate and incubated for up to 16 h at 37 °C. Cytopathic effects were observed under a light microscope at 100× magnification. (A) TPG culture medium (undiluted); (B) EHE-NE18 culture supernatant (1:16 dilution); (C) JIR325 culture supernatant (1:2 dilution); (D) NE18-M1 (*plc* mutant) culture supernatant (1:16 dilution). (B) And (D) show obvious cell rounding and detachment.

We previously showed that a *plc* mutant of the chicken isolate EHE-NE18 was still virulent in chickens [9]. Consequently, to determine if NE strains of C. perfringens produced extracellular toxins other than alpha-toxin, sterile filtered culture supernatants of strain EHE-NE18 and its *plc* mutant NE18-M1 [9] were incubated with various cell lines including Vero, DF1, HD11 and the chicken leghorn male hepatoma (LMH) cell line for 16 h. Cytopathic effects were observed with both EHE-NE18 and NE18-M1 supernatants on LMH cells at a dilution of up to 1/32 (Figure 1). By contrast, supernatant derived from EHE-NE18 but not NE18-M1 displayed cytopathic effects on Vero cells, indicating that this activity was caused by alpha-toxin. No cytopathic effects were seen after treatment of either DF1 or HD11 cells. Furthermore, no cytopathic effects were observed when LMH cells were incubated with supernatants from JIR325, a strain known to produce high levels of alpha-toxin but which does not cause NE in chickens [9]. These results indicated that the cytotoxicity for LMH cells was produced by a secreted component distinct from alpha-toxin. This cytopathic effect provided a biological assay that we used to identify the presence of toxin during its subsequent isolation and purification from EHE-NE18 supernatant fractions.

### Purification of a Cytotoxic Protein from EHE-NE18

**Figure 2:**
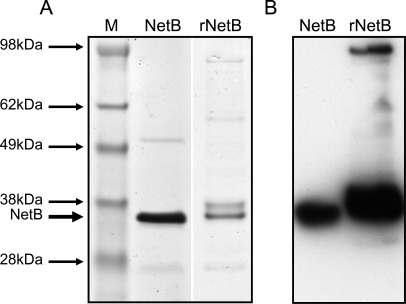
SDS-PAGE and Western Blot of Purified Native NetB and rNetB Toxin Proteins were separated by 4%–12% SDS-PAGE. Native NetB was prepared with Sepharose Q FF anion exchange resin and rNetB-toxin was purified on a nickel affinity column followed by gel filtration. SeeBlue Plus2 prestained marker (Invitrogen) was used as a size marker. (A) SDS-PAGE stained with coomassie blue: M, SeeBlue Plus2 marker; NetB, native NetB; rNetB, recombinant NetB. (B) SDS-PAGE transferred onto PVDF membrane and probed with rabbit polyclonal anti-rNetB antibody. Blots were developed with an ECL western blotting kit and the results recorded on autoradiographic film: NetB, native NetB; rNetB, recombinant NetB.

Ten ml of a 100× concentrated supernatant from a late log-phase culture of EHE-NE18 was loaded onto a Hi-Trap Mono-Q anion exchange column (GE Life Sciences) in 10 mM Tris-HCl buffer, pH 8.5. Proteins were eluted off the column stepwise, with increasing concentrations of NaCl (up to 1M). Analysis of the fractions in the LMH cytotoxicity assay revealed that only the flow-through fractions were positive (data not shown). These fractions were concentrated and analysed by SDS-PAGE and a major band was observed with a molecular mass of approximately 33 kDa (Figure 2A). N-terminal sequencing of this band identified the first 11 amino acids of the protein as S-E-L-N-D-I-N-K-I-E-L. Database searches failed to reveal any proteins with a similar sequence, indicating that a novel protein had been identified.

### Identification of a Novel Toxin Gene in EHE-NE18 Genomic Sequence

In an alternative but complementary approach, genomic sequencing was used in an attempt to identify the cytotoxin produced by EHE-NE18. The incomplete EHE-NE18 sequence obtained from 454 Life Sciences contained 3,470,244 bp and was assembled into 381 contigs (av size = 9108 bp). The sequence was analysed for coding potential using the algorithm GeneMarkS [14], which led to the identification of 3771 putative ORFs, with 3081 being longer than 99 amino acids. All 3771 EHE-NE18 ORFs were compared against all CDS features from the C. perfringens strain 13 [15], C. tetani [16] and C. acetobutylicum [17] genomes using BLAST [18], with a match threshold set at 20% identity. These analyses identified 309 EHE-NE18 ORFs with no significant matches to the C. perfringens strain 13 ORFs, 229 of which showed no significant match to either C. tetani or C. acetobutylicum ORFs*.* These unique ORFs were further analysed by PSORTB [19] and ProteomeAnalyst [20] to identify predicted extracellular proteins. One of these predicted extracellular proteins was found to have similarity to beta-toxin from C. perfringens (38% amino acid sequence identity) and therefore was designated as NetB (Necrotic Enteritis Toxin B-like, EU143239). A potential ribosome binding sequence was located seven bases upstream of the putative TTG start codon of *netB*, which encoded a putative 323 amino acid protein, with a signal peptide region of 30 residues as predicted by SignalP [21]. The molecular size of the mature NetB toxin, as calculated from the deduced amino acid sequence, was 33 kDa, which correlated with the size of the unknown cytotoxic protein purified from the EHE-NE18 supernatant. Moreover, the predicted N-terminal amino acids of the mature form of the NetB-toxin were identical to the amino acids determined by N-terminal sequencing of the EHE-NE18 cytotoxic protein.

**Figure 3:**
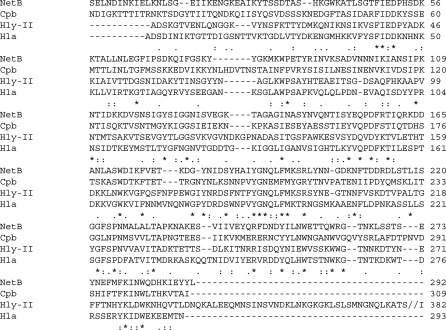
ClustalW Alignment of NetB ClustalW alignment of the toxins C. perfringens NetB (EU143239), C. perfringens beta-toxin (Cpb, AAA23284.1), B. cereus hemolysin II (Hly-II, NP_833256.1), and S. aureus alpha-toxin (Hla, NP_371687.1). Identical residues (*), conservative amino acid substitutions (:), and semi-conservative amino acid substitutions (.) are shown below the aligned sequences. To compact the figure the Hly-II sequence was shortened (//).

**Figure 4:**
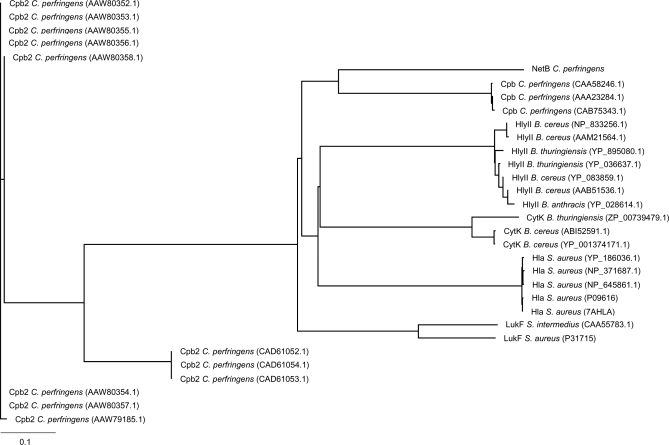
Phylogenetic Analysis of NetB Phylogenetic analysis of representative members of the “S. aureus pore-forming toxin family” with beta2-toxin (*Cpb2*) used as outliers. Clustal X with neighbour-joining bootstrapping (1000 interactions) was used to construct the tree. Toxins that were used included: Beta-toxin (Cpb) from C. perfringens (CAA58246.1, AAA23284.1, CAB75343.1); Hla from S. aureus (NP_371687.1, YP_186036.1, 7AHLA, P09616, NP_645861.1); HlyII from B. cereus (AAB51536.1, YP_083859.1, NP_833256.1, AAM21564.1); B. thuringiensis (YP_895080.1, YP_036637.1); B. anthracis (YP_028614.1); CytK from B. cereus (ABI52591.1, YP-001374171.1); B. thuringiensis (ZP_00739479.1); and LukF from S. aureus (P31715); S. intermedius (CAA55783.1). Beta2-toxin (*Cpb2*) from C. perfringens (CAD61052.1, CAD61053.1, CAD61054.1, AAW79185.1, AAW80352.1, AAW80353.1, AAW80354.1, AAW80355.1, AAW80356.1, AAW80357.1, AAW80358.1).

The deduced amino acid sequence of NetB displayed limited sequence similarity to a range of pore forming toxins including C. perfringens beta-toxin (38% identity), Bacillus cereus haemolysin II (29% identity) and the S. aureus alpha-hemolysin precursor (Hla; 30% identity) (Figure 3). Comparison of NetB against the CDD conserved domain database [22] showed that it contained a leukocidin domain, which is also present in beta-toxin, HlyII and Hla, indicating that NetB belongs to the Leukocidin/Hemolysin family of toxins. To further explore the relationship between NetB and other members of the S. aureus pore-forming toxin family, we used ClustalX and treeview to visualize the phylogenetic relationships (Figure 4). These data showed that the closest orthologues of NetB were the beta-toxin proteins from C. perfringens. However, although the NetB-toxin is most similar to C. perfringens beta-toxin the level of identity is relatively low and NetB resides on its own deep branch (Figure 4) indicating that it should not be considered as a beta-toxin variant but as a distinct toxin.

### Both the Native and Recombinant NetB Proteins Are Cytotoxic for LMH Cells

The *netB* gene was amplified from EHE-NE18 using PCR, cloned into the expression vector pDest41BA (Table 1) and recombinant NetB produced in E. coli. Expression from pDest41BA results in fusion of the recombinant protein with both an N-terminal NusA solubility tag and an N-terminal 6xHis tag. The expressed NetB fusion protein was purified on a nickel affinity column and the NusA and 6xHis fusion tags removed by TEV cleavage. SDS-PAGE indicated that the purified and cleaved protein (designated rNetB) ran at an identical molecular size to the native protein purified from C. perfringens (Figure 2A). Furthermore, antibodies raised in rabbits against the rNetB protein recognised both native and recombinant NetB (Figure 2B).

**Table 1:**
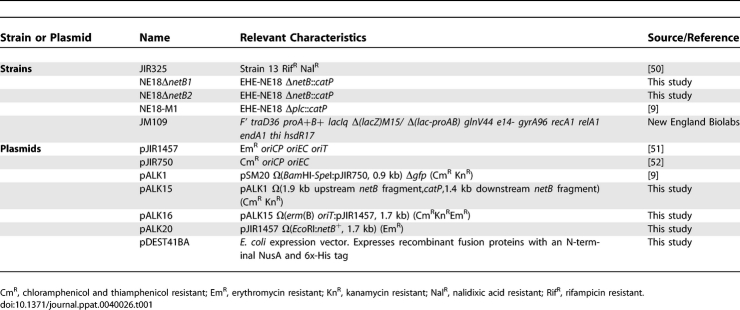
Bacterial Strains and Plasmids

**Figure 5:**
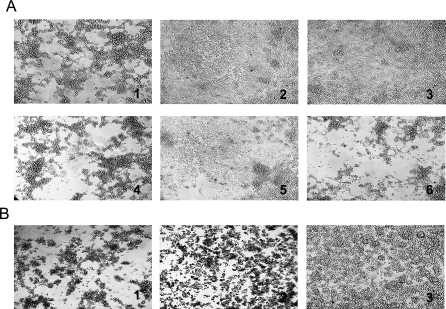
Cytotoxic Activity of Supernatants from EHE-NE18, EHE-NE18Δ*netB1*, and Complemented Mutants The LMH cells were cultured until 70% confluence in 24 well plates coated in 0.2% gelatine and grown in EMEM medium at 37 °C. (A) Cytotoxicity assays. Culture supernatant was added to the medium and incubated for up to 16 h at 37 °C. (1) EHE-NE18 (1:16 dilution); (2) NE18Δ*netB1* (1:2 dilution); (3) NE18Δ*netB1*(pJIR1457) (shuttle plasmid)(1:2 dilution); (4) NE18*ΔnetB1*(pALK20) (*netB^+^* complementation plasmid) (1:16 dilution); (5) TPG culture medium (undiluted); (6) Purified rNetB (0.5 μg). (B) Neutralisation of NetB cytotoxicity. Pre-immune sera and antisera raised against rNetB were incubated with 0.5 μg/ml of purified native NetB-toxin (1:20) for 1 h at room temperature. Treated and non-treated samples where added to LMH cells and incubated for 16 h at 37 °C. Cytopathic effects were observed under the light microscope at 100× magnification. (1) NetB positive control; (2) NetB pretreated with pre-immune rabbit sera; (3) NetB pretreated with rabbit anti-rNetB antiserum.

To determine if rNetB was active, LMH cells were treated with the purified protein (Figure 5A, panel 6). Cytopathic effects were observed at similar dilutions to purified native NetB. In addition, preincubation of both rNetB and NetB with the antiserum raised against the *E. coli-*derived rNetB protein neutralized both toxins in the cytotoxicity assay (Figure 5B and data not shown) while pre-treatment with pre-immune sera did not neutralize either toxin.

### Osmotic Protection and Estimation of the NetB Pore Size in LMH Cells

**Figure 6:**
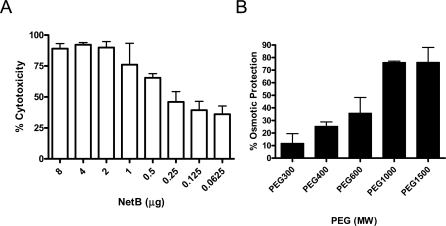
Lactate Dehydrogenase Cytotoxicity Assay of LMH Cells Treated with NetB and NetB Pore-size Estimation (A) Purified NetB was added to the tissue culture medium with two-fold dilutions up to 62.5 ng of toxin and incubated with LMH cells for 4 h at 37 °C. The percentage of total LDH released in the supernatant was measured with a Cyto-Tox (Promega) kit and used as an indicator of cytolysis. Each dilution was assayed in triplicate in three independent experiments. (B) LMH cells were incubated with 0.5 μg/well protein and 25 mM PEGs with different molecular sizes for 4 h, and the extent of cellular lysis was determined by measuring the amount of LDH released. The results are presented as a percentage of the control (cells without any PEG). The values are averages of triplicate assays in three experiments with error bars representing SEM.

Since bioinformatics analysis suggested that NetB may be a pore-forming toxin, cytotoxicity assays were carried out in the presence of polyethyleneglycol (PEG) molecules of varying sizes to allow estimation of the NetB pore size in the LMH cell membrane. LMH cells were incubated with purified toxin and lactate dehydrogenase (LDH) release into the supernatant was measured as an indicator of cytolysis using the Cyto-Tox (Promega) kit (Figure 6A). The NetB-induced LMH cytotoxicity was not significantly inhibited by PEG 300 (hydrodynamic diameter of 1.16 nm), was partially inhibited by PEG 400 and PEG 600 (hydrodynamic diameters of 1.36 nm and 1.6 nm, respectively), and was strongly inhibited by PEG 1000 (hydrodynamic diameter of 1.8 nm) and PEG 1500 (hydrodynamic diameter of 2.4 nm) (Figure 6B). These results indicate that NetB forms a hydrophilic pore in the cell membrane with a functional diameter of approximately 1.6–1.8 nm.

### Most NE-causing C. perfringens Strains Contain the *netB* Gene and Produce NetB-toxin

The presence of the *netB* gene in NE-causing and non-NE-derived strains of C. perfringens was investigated by PCR using *netB*-specific primers. Of the 18 poultry-derived NE C. perfringens strains tested, 14 were positive for *netB* (data not shown) although three of the four *netB* negative isolates were from the same NE outbreak and are most likely from the same clonal population. No *netB*-specific product was observed in any of the 32 non-NE-derived strains, which included C. perfringens type A, B, C and D strains isolated from cattle, sheep, pigs and humans. The production of NetB toxin in the *netB*-positive strains was confirmed by Western blotting of culture supernatants using rabbit antiserum against rNetB. There was an absolute correlation between the *netB*-PCR and the NetB-Western blots (data not shown) with a single band visualised for each positive strain at the appropriate size similar to native NetB. None of the strains that were negative in the PCR assay for *netB* produced an immunoreactive NetB band. Moreover, no cytopathic effect was observed when culture supernatants from the four *netB*-negative NE strains were incubated with LMH cells (data not shown).

### Construction of a *netB* Mutant of EHE-NE18

**Figure 7:**
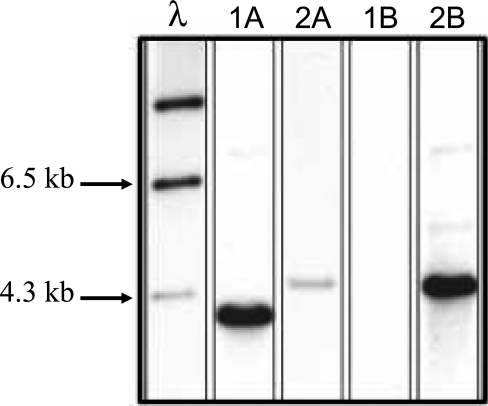
Southern Hybridization Analysis of *Hin*dIII-digested Genomic DNA DNA from EHE-NE18 (1) and NE18Δ*netB1* (2) was probed with DIG-labelled DNA specific for the *netB* (A) and *catP* (B) genes as shown. Appropriate lambda DNA size markers are arrowed and labelled at the left.

To determine the role of NetB in the pathogenesis of NE in chickens, a *netB* mutant was isolated. The Δ*netB* suicide plasmid pALK16 (Table 1) was constructed and used to transform strain EHE-NE18 to thiamphenicol resistance. Two independently derived *netB* mutants, NE18Δ*netB1* and NE18Δ*netB2*, were isolated from five separate transformation experiments. Their genotypes were confirmed by PCR (data not shown) and Southern blot analysis (Figure 7). Genomic DNA from EHE-NE18 and NE18Δ*netB1* was digested with *Hin*dIII and analysed separately with DIG-labelled *catP* and *netB* specific probes. As expected, the *netB* probe bound to DNA from both the wild-type and mutant strains, hybridising to the expected 3.8-kb genomic DNA fragment in the wild-type strain and to a 4.4-kb fragment in the NE18Δ*netB1* mutant. The hybridising NE18Δ*netB*1 fragment was less intense than the hybridising wild-type fragment as most of the sequence present in the probe had been deleted in the mutant strain. In NE18Δ*netB1* the *catP* probe hybridized to a band of the same size as the *netB* reactive band, but did not bind to EHE-NE18 DNA. These data confirmed that both mutants were derived from double reciprocal crossover events between pALK16 and the *netB* region in EHE-NE18. Similar results were obtained with the second independently derived *netB* mutant (NE18Δ*netB2*). Culture supernatants from the *netB* mutant strains showed no cytotoxicity in the LMH assay (Figure 5A) but were capable of alpha-toxin and perfringolysin O expression as demonstrated by the presence of zones of precipitation or hemolysis on egg yolk agar and sheep blood agar, respectively (data not shown).

To confirm that the loss of cytotoxicity was associated with the mutation in *netB*, both mutants were complemented *in trans* with the multicopy plasmid, pALK20 (Table 1), which contained a wild-type copy of the *netB* gene cloned into the shuttle vector, pJIR1457 (Table 1), These plasmids were introduced separately into the mutant strains and the resultant strains assayed for their cytotoxicity Cytotoxic activity was restored when the *netB* mutant strains were complemented with pALK20 but not with pJIR1457 (Figure 5A). Therefore, NetB is responsible for the cytotoxic activity of EHE-NE18 supernatants on LMH cells.

### The *netB* Mutants Do Not Produce NE in a Chicken Disease Model

**Figure 8:**
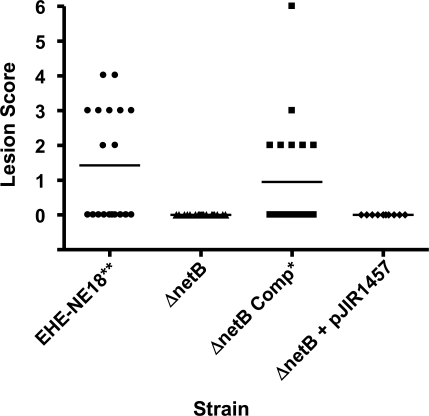
Virulence of C. perfringens Strains in the NE Challenge Model The lesion scores of individual 24-day-old broiler chickens challenged with different C. perfringens strains are shown. Each group consisted of ten birds. The solid horizontal bars represent the average lesion score in each group. Intestinal lesions in the small intestine (duodenum to ileum) were scored as previously reported [9]: 0, no gross lesions; 1, thin or friable walls; 2, focal necrosis or ulceration (one to five foci); 3, focal necrosis or ulceration (six to 15 foci); 4, focal necrosis or ulceration (16 or more foci); 5, patches of necrosis 2–3 cm long; 6, diffuse necrosis typical of field cases. The results are from two separate trials. The strains tested are as follows: EHE-NE18, wild-type; Δ*netB*, NE18Δ*netB1*; Δ*netB* Comp, NE18Δ*netB1*(pALK20); Δ*netB1* + pJIR1457, NE18Δ*netB1*(pJIR1457). One-tailed, nonparametric t-test analysis of the challenge (EHE-NE18) and complemented mutant derivatives against NE18Δ*netB1* showed a statistical difference (**, *p* < 0.01 and *, *p* < 0.05) but no statistical significance was observed between the mutant and the plasmid control.

To determine if the *netB* mutant could cause NE, groups of 10 Ross 308 broiler chickens were challenged with wild-type strain*,* the isogenic NE18Δ*netB1* mutant, or the complemented NE18Δ*netB1* strains in a NE disease induction model. No NE lesions were detected in birds infected with the *netB* mutant or the mutant complemented with the vector plasmid pJIR1457. By contrast, significant levels of disease were detected in birds infected with the wild-type parent strain (*p* < 0.01) or the strain complemented with the *netB^+^* plasmid pALK20 (*p* < 0.05) (Figure 8). Similar results were obtained in independent virulence trials. Bacteria were cultured from the intestines of birds from all groups and with one exception only the infecting strain was isolated from the lesions. In one of the trials, two birds in the *netB* mutant challenge group showed a small number of lesions typical of NE. However, no thiamphenicol resistant C. perfringens were isolated from these birds and it was concluded that these lesions were likely to have been caused by environmental or endemic C. perfringens isolates and therefore are not included in our analysis.

## Discussion

In this paper we report the identification and initial characterization of NetB, a novel C. perfringens toxin. This toxin appears to be carried only by NE-causing C. perfringens type A strains as none of the 32 non-NE-derived strains tested either produced the toxin or carried the *netB* gene. The *in vivo* analysis of an isogenic *netB* mutant and its complemented derivative provided unequivocal evidence that NetB is an essential virulence factor in the pathogenesis of NE in chickens. When the *netB* gene was insertionally inactivated by a double crossover event the resultant mutant was avirulent in the NE disease model. Complementation with the wild-type *netB* gene restored the ability to cause NE in chickens, fulfilling the requirements of molecular Koch's postulates. Importantly, the gross lesions observed in the animal model were consistent with NE lesions previously reported from experimental and field cases of the disease [6,7,9,23,24]. While, in this study, we only tested strains from chickens suffering NE and NetB was not present in non-NE strains, further more extensive epidemiological studies need be carried out to determine the international distribution of this new toxin and its correlation with disease.

For many years alpha-toxin was believed to be the main virulence factor involved in NE pathogenesis in chickens. However, this hypothesis was based on studies that used culture supernatants to reproduce the disease [6] even though the supernatants could potentially contain many secreted proteins. Furthermore, subsequent studies [8,25] that used antibodies prepared against culture supernatants or partially purified toxin preparations did not explore the possibility that toxins other than alpha-toxin might be produced by the C. perfringens strains. The identification of NetB, together with our recent observation that alpha-toxin negative C. perfringens mutants remain virulent [9], adds further weight to the proposal that alpha-toxin plays only a minor role in NE disease pathogenesis.

In other studies the presence of beta2-toxin in C. perfringens type A strains has been associated with enteric diseases in human and animals [26–28]. The detailed analysis of these isolates has revealed that a high proportion of strains from avian sources contain an atypical beta2-toxin [29]. While this gene is present in a high proportion of non-porcine C. perfringens isolates it remains unclear if it is involved in enteric diseases of other animals including chickens. Importantly, *cpb2* positive strains can be found in both healthy and diseased chickens; its presence does not correlate with disease [30].

The mature NetB toxin was similar in molecular size to mature beta-toxin (33.2 kDa vs 34.8 kDa). However, the two toxins have limited sequence identity (38%) and phylogenetic analysis indicated that NetB was clearly a distinct toxin that is not a member of the beta-toxin clade (Figure 5B). However, the presence of conserved amino acid residues in NetB and beta-toxin suggested that NetB may also act as a pore forming toxin [13]. NetB displays many of the conserved residues found in other pore-forming toxins of the S. aureus alpha-toxin family [31]. Site-directed mutagenesis has been used to characterize many amino acids critical for function in C. perfringens beta toxin and S. aureus alpha toxin [32,33] and NetB contains many of these key residues including R200 (R212 from beta-toxin). The corresponding residue in S. aureus alpha-toxin (R200) is important for binding and oligomerization of the protein as well as for hemolysis [34]. Mutation of the beta-toxin residue Y203 (Y191 in NetB) to phenylalanine resulted in a 2.5-fold increase in the LD_50_. The corresponding residue in S. aureus alpha-toxin (Y191) is located at the predicted membrane binding surface of the protein [35]. Mutation of the beta-toxin residue D167 (D156 in NetB) resulted in a complete loss of functional protein expression, suggesting that this region participates in crucial protomer-protomer interactions as well as being important for conformational rearrangements involved in multimer formation in the membrane bound form [35]. Since NetB aligns at many of these specific residues essential for beta-toxin and S. aureus alpha-toxin function we postulate that NetB is likely to be a pore-forming toxin.

We have shown that NetB induced morphological changes in LMH cells, resulting in significant rounding and cell lysis. Since these morphological changes and LDH release were blocked by PEG1000 and PEG1500, it was likely that these changes were caused by toxin-dependent formation of pores in the plasma membrane. Osmotic stabilizers such as PEG can inhibit the lysis of target cells if they are unable to pass through the pore generated by a pore-forming toxin [36]. This observation has been used to estimate the size of the pore formed by various other pore-forming toxins, including the Xenorhabdus nematophila fimbrial shaft protein (MrxA) [37], the E. coli hemolysin [38], the C. septicum alpha-toxin [36] and the Vibrio metschnikovii cytolysin [39]. Based on the estimated Stokes radii for various PEG molecules [40] the results obtained here suggest that NetB forms a hydrophilic pore with a functional diameter of 1.6–1.8 nm in the cell membrane [41]. This NetB pore diameter is slightly larger to the pore size predicted for beta-toxin (1.36–1.6 nm) in HL 60 cells [12].

Although a high proportion (14/18) of NE strains contained *netB* and expressed the NetB toxin we identified four NE strains that were *netB*-negative. These strains were isolated from chickens suffering NE and were able to cause NE in our experimental model (data not shown) however, three of these strains were isolated from the same NE outbreak and may represent the same clonal population. These data indicate that the presence of NetB may not be essential for the disease process in all C. perfringens isolates. In EHE-NE18 it is clear that production of NetB toxin is required for disease. However, in the virulent *netB*-negative strains other, as yet unidentified factors, may be sufficient for disease production. Indeed, there may be distinct classes of NE-causing C. perfringens strains and we are currently analysing these other strains for novel toxins.

In conclusion, we have identified a novel C. perfringens pore-forming toxin which we designated NetB. While it is possible that NE may result from the interaction of several toxic molecules it is clear from the evidence provided here that NetB is a critical C. perfringens EHE-NE18 virulence factor and to our knowledge is the first virulence factor unequivocally shown to be associated with NE in chickens. The identification of this important toxin opens significant opportunities for the development of novel vaccines against NE in poultry and these studies are currently underway in our laboratories.

## Materials and Methods

### Bacterial strains and growth conditions.

Bacterial strains and plasmids are described in Table 1. C. perfringens was grown in tryptone-proteose peptone glucose (TPG) [42], fluid thioglycollate broth (FTG, Beckson, Dickinson and Company), TSC agar (Oxoid), egg-yolk agar (EYA) [43] and Brain Heart Infusion agar with 5% sheep blood (SBA). Agar cultures were grown at 37 °C in an atmosphere containing 10% H_2_, 10% CO_2_ and 80% N_2_. C. perfringens media were supplemented with thiamphenicol (5 μg/ml) or erythromycin (50 μg/ml) as required. E. coli strains were derivatives of JM109 and were grown in LB broth or agar under aerobic conditions at 37 °C. E. coli media were supplemented with ampicillin (100 μg/ml), erythromycin (150 μg/ml) or chloramphenicol (30 μg/ml) as required.

### Genomic sequencing and bioinformatics of EHE-NE18.

The genomic DNA sequence of C. perfringens EHE-NE18 was determined by 454 Life Science Corp. GeneMarkS was used to predict possible open reading frames [14]. Signal peptide prediction was performed using the SignalP v 3.0 program [21]. Sequences homologous to the deduced amino acid sequence were searched using the gapped BLAST program [18]. A multiple sequence alignment was executed using the CLUSTAL W program [44]. ClustalX [45] with Neighbour-Joining bootstrap (1000 iterations) analysis was used to construct a phylogenetic tree of NetB and related toxins. The tree was visualized using TreeView [46].

### Native NetB production and purification.

EHE-NE18 was grown in TPG broth to a turbidity of 0.6 at 600nm. Culture supernatant (3 L) was obtained by centrifugation at 18 000 *g* for 15 min at 4 °C. The supernatant was concentrated 5× using ultrafiltration (Amicon 8400) through a 10kDa membrane (DIAFLO® YM10–76 mm, Amicon) and supernatant proteins precipitated by overnight incubation in 40% (w/v) (NH_4_)_2_SO_4_ at 4 °C overnight followed by centrifugation at 18 000 *g* for 2 h at 4 °C. The precipitate, containing the toxin, was resuspended in 30 ml of PBS and dialysed against 10mM Tris-HCl buffer, pH 8.5 (5 L) for 48 h at 4 °C with 5 buffer changes. Proteins were separated by Sepharose Q FF (GE) anion exchange chromatography in 10 mM Tris-HCl buffer (pH 8.5) and the flow through collected. The NetB protein (detected at approximately 33 kDa) was transferred to a PVDF membrane (PALL) in CAPS buffer (Sigma) by the method of Towbin *et al.*[47] and the sequence of the 11 N-terminal amino acid residues determined by Edman degradation on a Procise 492 protein sequencer (Applied Biosystems).

### Purification of recombinant NetB and generation of rabbit anti-rNetB serum.

The *netB* gene was amplified by PCR, cloned into the Gateway™ entry vector pENTR/SD/D-TOPO (Invitrogen) and transferred into the modified Gateway™ expression vector pDest41BA (Table 1). The recombinant fusion protein was purified on a nickel affinity column followed by gel filtration on Superdex 200. Peak fractions were pooled and the 6xHis tag cleaved using TEV protease and reloaded onto a nickel column to remove both uncleaved protein and the TEV protease. Recombinant protein (∼1.3 mg) was sent to Chemicon (Chemicon-Millipore) for antibody production in rabbits. The rNetB-toxin antiserum was used in Western blot analysis of C. perfringens strains and for neutralization studies. In the latter studies antisera raised against rNetB was incubated with semi-purified native NetB toxin (1:20) for 1 h at room temperature before being tested in the LMH cytotoxicity assay. Pre-immune sera and untreated toxin were used as controls.

### Western blot analysis of C. perfringens strains*.*

C. perfringens strains were grown in TPG broth to a turbidity at 600nm of 0.6. Culture supernatants were obtained by centrifugation at 18 000 *g* for 10 min and separated by SDS-PAGE (NuPAGE ® Novex 4–12% Bis-Tris gel, Invitrogen) in MES SDS running buffer (NuPAGE ®MES SDS Running Buffer, Invitrogen). Proteins were transferred onto PDVF (PALL) membranes and probed with rabbit polyclonal anti-rNetB antiserum. Blots were developed with an ECL Western Blotting kit (Amersham Biosciences) and the results recorded on autoradiographic film.

### Construction of defined *netB* mutants of EHE-NE18.

DNA manipulations were carried out according to standard techniques [48]. All amplified products were cloned into the pGEM®-T Easy vector system (Promega) and subsequently sub-cloned as required. The marked, partial deletion, suicide plasmid, pALK16, was constructed by cloning fragments of the *netB* gene and surrounding regions on either side of the *catP* cassette in pALK1 [9], which resulted in an 541 bp internal deletion within the *netB* gene. First, a 1490 bp *Mfe*I-*Spe*I fragment amplified using oligonucleotides AKP60 (5′-GCAATTGGCAAGATCATAAAATAGAA-3′) and AKP61 (5′-CCCTAGGTATTTTCTTATCGCTACTTG-3′) was directionally cloned into the *Eco*RI-*Spe*I sites of pALK1, followed by cloning of a 1937 bp *Bam*HI-*Nhe*I fragment amplified using oligonucleotides AKP58 (5′-GGGATCCAATTGTAAACATTCCTGATA-3′) and AKP59 (5′-AGCTAGCTATTATTATTTACATCAGCACTTT-3′) into the *Bam*HI-*Nhe*I sites of the resultant plasmid. Finally, the *erm*B gene and the *oriT* region were amplified from pJIR1457 and blunt-end cloned into the *Sma*I site. The final suicide plasmid pALK16 was introduced into C. perfringens strain EHE-NE18 as described previously [49]. After growth at 37 °C on TSC supplemented with thiamphenicol, colonies were patched onto TSC supplemented with erythromycin to confirm that a double crossover event had occurred. The colonies that were thiamphenicol resistant and erythromycin sensitive were selected for further analysis. Genomic DNA was prepared and PCR and Southern blot analysis was used to confirm that the mutants were derived from double crossover events within the *netB* gene region. The primer pairs of AKP53 (5′-AAGTGCTGCAGTCCAATTATG-3′) and AKP8 (5′-TGATTCCTATTTTTACTATGG-3′), AKP48 (5′-TTGCTCTAGCAAGCCCATTC-3′) and AKP7 (5′-CTTTTCCTTTATTTGTGTGAT-3′), and AKP47 (5′-CCGCTTCACATAAAGGTTGG-3′) and AKP49 (5′-CTGTTCCATTCCCTTGAGGA-3′) were used for confirmation of correct insertion within the *netB* region, and Southern blots were probed with labelled *netB* and *catP* gene fragments. The complementation plasmid, pALK20, was constructed by cloning the wild-type *netB* gene into the C. perfringens shuttle vector pJIR1457. The complementation plasmid pALK20 and the vector pJIR1457 were introduced into confirmed mutants by electroporation [49] and erythromycin resistant transformants selected.

### Southern hybridization analysis.

Genomic DNA of wild-type and *netB* mutant strains was digested with *Hin*dIII, separated by gel electrophoresis on a 0.8% agarose gel and transferred to a nylon membrane (Hybond N; Amersham). The blots were hybridized with DIG-labeled PCR amplified probes as specified by the manufacturer (Roche). The probes were either a 410-bp *catP*-specific product amplified from pJIR750 or a 563-bp *netB* product amplified from EHE-NE18 genomic DNA. Hybridization was detected using a chemiluminescence detection system (Roche).

### Detection of the *netB* gene in various strains.

The presence of the *netB* gene in various strains of C. perfringens was investigated by PCR. A single colony of each strain was suspended in 100 μl distilled water, boiled for 10 min and then centrifuged at 10 000 *g* for 10 min. The supernatants were collected and used as the template for PCR. PCR was performed in a 25 μl reaction mixture containing: 1x PCR buffer (Mg^2+^ free, Promega); 2.5 mM MgCl_2_; 0.2 mM dNTP mixture; 2.5 units of Go *Taq* DNA polymerase (Promega); 50 pM of primers (AKP78 5′-GCTGGTGCTGGAATAAATGC-3′and AKP79 5′-TCGCCATTGAGTAGTTTCCC-3′); and 5 μl of template solution. The following conditions were used: denaturation at 94 °C for 2 min; 35 cycles of denaturation at 94 °C for 30 s; annealing at 55 °C for 30 s; and extension at 72 °C for 1 min; with the final extension step at 72 °C for 12 min. PCR products were analysed by electrophoresis on 1.5 % agarose gels.

### Cell lines and cytotoxicity assay.

The chicken hepatoma cell line LMH (ATCC CRL-2117) and the Vero African green monkey kidney cell line (Vero, ATCC CCL-81) were maintained in Earl's minimum essential medium (EMEM) supplemented with L-glutamine, 10% fetal calf serum (FCS), 10mM 4-(2-hydroxyethyl)-1-piperazineethanesulfonic acid (HEPES), 100 U/ml penicillin, 100 μg/ml streptomycin and 100 μg/ml Fungizone (LMH cells also require 0.2% gelatine for adherence to surfaces). Chicken embryo fibroblast cells (DF-1, ATCC CRL-12203, a cell line derived from East Lansing ELL-0 chicken eggs) and chicken macrophage-like cells (HD11) were grown in DMEM supplemented with 10% FBS, 100 U/ml of penicillin and 100 μg/ml of streptomycin. All cell lines were incubated in a humidified environment of 5% CO_2_ at 37 °C. To test for cytotoxicity cell lines were cultured to 70% confluence in 24 well plates (Nunc) and grown in their respective growth medium at 37 °C. Culture supernatant or semi-purified toxin was added to the medium and diluted in two-fold steps across the plate, to a dilution of 1:512, and incubated for up to 16 h at 37 °C. Both visual cytopathic effects and lactate dehydrogenase release in the supernatant was used as an indicator of cytolysis. The Cyto-Tox (Promega) kit was used to measure LDH release and the results expressed as percentage cytotoxicity.

### Pore size estimates.

The size of the pore generated by NetB on LMH cells was determined by osmotic protection of toxin-treated cells with PEG preparations of various molecular sizes (PEG 300 - PEG 1500) [37]. LMH cells were cultured in 96-well tissue culture plates (Nunc) in 200 μl of EMEM culture medium. Osmotic protection of the cells by PEG (25 mM) was performed in the presence of 0.5 μg of NetB per well, which caused 50% lysis of the cells in 4 h. The experiment was repeated three times, and the values reported represent the mean ± the standard deviation of triplicate samples from one representative experiment. Percent protection was calculated based on the amount of LDH released where the LDH release in the absence of PEG was taken as 100% and in the absence of NetB was taken as 0%. Pore-size estimation was determined using the calculated Einstein-Stokes radius for each of the PEG molecules used [40,41].

### *In vivo C. perfringens* challenge model.

The NE disease induction model was performed as previously described [9]. Briefly, commercial 1-day-old Ross 308 broiler chickens were fed an antibiotic-free chicken starter diet containing 20% protein for 13 days. On day 14 feed was changed to a wheat-based feed containing 50% fishmeal. On day 20, feed was withdrawn and each bird was orally challenged with 1.5 ml of C. perfringens culture (10^9^ to 10^10^ CFU). On day 21 birds were again orally challenged and feed contaminated with C. perfringens was administered. C. perfringens strains were grown in FTG broth with the addition of 2% soluble starch and 1.5% thiopeptone and incubated at 37 °C for 14 h. Groups of 10 chickens were kept in adjacent separate pens in an animal isolation facility. On day 24, chickens were euthanased with inhaled carbon dioxide gas and their small intestines (duodenum to ileum) examined for gross necrotic lesions. Intestinal lesions in the small intestine (duodenum to ileum) were scored as follows: 0 = no gross lesions; 1 = thin or friable walls; 2 = focal necrosis or ulceration (1–5 foci); 3 = focal necrosis or ulceration (6–15 foci); 4 = focal necrosis or ulceration (16 or more foci); 5 = patches of necrosis 2–3 cm long; 6 = diffuse necrosis typical of field cases. C. perfringens was recovered from the intestines of killed chickens by culturing on TSC agar. All animal experiments were assessed, approved and monitored by the Australian Animal Health Laboratories animal ethics committee.

## Supporting Information

### Accession Numbers

The GenBank (http://www.ncbi.nlm.nih.gov/Entrez/) accession numbers in this study are: NetB from C. perfringens: EU143239; Beta-toxin *C. perfringens*: CAA58246.1, AAA23284.1, CAB75343.1; Hla from S. aureus: NP_371687.1, YP_186036.1, 7AHLA, P09616, NP_645861.1; HlyII from B. cereus: AAB51536.1, YP_083859.1, NP_833256.1, AAM21564.1; *B. thuringiensis:* YP_895080.1, YP_036637.1; B. anthracis: YP_028614.1; CytK from *B. cereus:* ABI52591.1, YP-001374171.1; *B. thuringiensis:* ZP_00739479.1; LukF from S. aureus: P31715; *S. intermedius:* CAA55783.1; Beta2-toxin (*Cpb2*) from C. perfringens: CAD61052.1, CAD61053.1, CAD61054.1, AAW79185.1, AAW80352.1, AAW80353.1, AAW80354.1, AAW80355.1, AAW80356.1, AAW80357.1, AAW80358.1.
